# Rituximab-Induced Serum Sickness Overlapping With Anaphylaxis: A Case Report

**DOI:** 10.7759/cureus.38333

**Published:** 2023-04-30

**Authors:** Adrien Cottu, Tarik Chaara, Weniko Caré, Hélène Vanquaethem, Hubert Nielly

**Affiliations:** 1 Internal Medicine, Université Pierre et Marie Curie Paris 6, Sorbonne Universités, Paris, FRA; 2 Internal Medicine, Bégin Military Teaching Hospital, Saint-Mandé, FRA; 3 Toxicology, Hôpital Fernand Widal, Assistance Publique – Hôpitaux de Paris, Paris, FRA

**Keywords:** immune complexes, biotherapy allergy, anaphylaxis, rituximab, serum sickness

## Abstract

Serum sickness (SS) and anaphylaxis are well-documented complications of rituximab (RTX) infusions. While the first presents usually as a triad of fever, rash, and arthralgias occurring seven to 14 days after infusion, the second presents as a sudden onset of hemodynamic instability, bronchospasm, and a pruritic erythematous rash, occurring within the first few hours after infusion. We present here a case of serum sickness associated with anaphylaxis five days after the first infusion of a second course of RTX. Only eight cases of rituximab-induced serum sickness (RISS) associated with hemodynamic instability have been reported. We describe the first case of proven anaphylaxis with an elevated tryptase serum level occurring in conjunction with RISS, six days after a third RTX infusion.

## Introduction

Monoclonal antibodies can cause type I and type III hypersensitivity reactions (HSR). Anaphylaxis is a type I HSR most often triggered by specific IgE binding to the responsible antigen. It involves the release of tryptase, histamine, leukotrienes, and prostaglandins from mast cells and basophils. Clinical manifestations include a sudden pruritic erythematous rash, bronchospasm, and hemodynamic instability occurring within minutes to hours of exposure [1]. Serum sickness (SS) is a type III HSR mediated by circulating immune complex (IC) deposits in target tissues. Clinical manifestations include the triad of fever, rash, and arthralgia, usually occurring seven to 14 days after exposure [2,3].

Here we present a case of rituximab-induced serum sickness (RISS) overlapping with anaphylaxis. To our knowledge, this is the first described case of proven anaphylaxis with an elevated tryptase serum level occurring in conjunction with RISS.

## Case presentation

An 86-year-old woman with no history of allergy or auto-immune disease presented with warm autoimmune hemolytic anemia (wAIHA) with a positive direct antiglobulin test for IgG and complement component (c)3, and negative antinuclear antibodies. She was only taking metformin, gliclazide, and vildagliptin for type II diabetes. The wAIHA revealed a marginal zone lymphoma diagnosed by blood immunophenotyping and bone marrow biopsy. The lymphoma was otherwise not symptomatic. She received oral prednisone tapered to 5mg/day over six months and stopped after eight months. Due to the first relapse, she received prednisone 1mg/kg/day associated with rituximab (RTX) 1000mg on day 1 and 1000mg on day 15. The first infusion was temporarily slowed because of an increase in body temperature (38°C) with chills despite accurate premedication with 60mg methylprednisolone and 5mg dexchlorpheniramine. No adverse event occurred with the second slow flow infusion. Steroids were stopped after one month.

One year later, the wAIHA relapsed and the patient received prednisone 1mg/kg/day. A second course of RTX (four infusions of 375mg/m2/week for four weeks) was started with the same premedication protocol. Five days after the first infusion, the patient presented with fever, arthralgia, and fixed urticarial papules on the face, chest, arms, and back. Antihistamines were ineffective. Approximately 15 hours later, the patient presented with hypotension to 70/40mmHg and mottling which persisted after a 1.5 L venous infusion of 0.9% sodium chloride. She was referred to the intensive care unit. No bronchospasm or clinical/biological signs of infection were observed. Echocardiography was normal. The plasma C-reactive protein (CRP) and the tryptase serum level were increased and total hemolytic complement (CH50) and C4 were consumed. No circulating IC was found, but anti-RTX IgG antibodies were very high (Table 1).

**Table 1:** Laboratory investigations at initial workup RTX: Rituximab, C: Complement component, CH50: Total hemolytic complement

Biological assay	Value	Reference range
C-reactive protein	530mg/L	Normal <5mg/L
Tryptase	26.3µg/L	Normal <13.5µg/L
C3	0.97g/L	Normal >0.90g/L
C4	0.06g/L	Normal >0.10g/L
CH50	12.5IU/L	Normal >31.6IU/L
Anti-RTX IgG antibodies	>100ng/mL	Normal <5ng/mL

**Figure 1:**
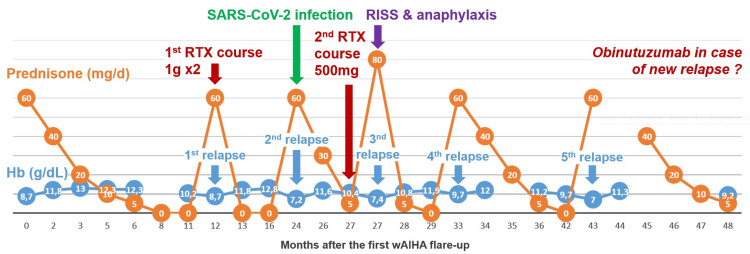
Evolution of wAIHA and specific treatments Hb: Hemoglobin; RISS: Rituximab-induced serum sickness; RTX: Rituximab; wAIHA: Warm autoimmune hemolytic anemia

Hemodynamic instability and urticaria resolved immediately after the start of epinephrine administration, but epinephrine infusion had to be maintained for two days up to 0.7mg/h. Methylprednisolone was started at 1mg/kg/day and tapered over one week. The triad of fever, rash, and arthralgia resolved in four days. One month later, the hemoglobin level was almost normalized, and the tryptase level was 9.6µg/L. Overall, she received 2,500mg of RTX, i.e., two 1000mg infusions during the first course, and only one 500mg infusion during the second course, which was stopped after this episode. Prednisone was tapered to 5mg/day and then stopped. The wAIHA relapsed twice after prednisone discontinuation. It was restarted both times with efficiency (Figure 1). At the end of the follow-up, alternative anti-cluster of differentiation (CD)20 antibodies such as obinutuzumab were discussed for future relapses.

## Discussion

This case presents clinical and biological features of both type I and III HSRs. Serum sickness was suggested by the subacute onset of fever, arthralgias, fixed and antihistamines-resistant rash, elevated plasma CRP level, complement consumption, and positivity of anti-RTX IgG antibodies [2-4]. Anaphylaxis was suggested by the acute onset of hemodynamic instability, resolved by treatment with epinephrine, and by a transient increase in serum tryptase levels [1]. A delayed cytokine-release syndrome (CRS) could also explain the fever, pain, rash, and shock, but not the biological abnormalities. As type I HSR and CRS clinical and biological presentations can sometimes be hard to distinguish, we believe that a CRS could partially participate in these symptoms. Cytokines dosage (IL-6, IL-1, IL-8, IL-10, tumor necrosis factor-alpha (TNF-α), interferon‐gamma (IFN-γ)) is lacking to argue this hypothesis [5]. Their normality could have helped exclude a CRS. On the other hand, their elevation could not have confirmed a CRS as they are also elevated in anaphylaxis [6].

A French pharmacovigilance database study from 1998 to 2016 reported hypotension in six out of 37 RISS cases (16.2%). Unfortunately, patient characteristics and administered treatments, such as vasopressor therapy, were not detailed [3]. In the international literature, there are four cases reports of RISS followed later by anaphylaxis [4,7-9], but only two cases of RISS occurring simultaneously with hypotension requiring vasopressor therapy (but without tryptase assay) [10,11], as shown in Table 2. Our case is the first report of RISS occurring in conjunction with anaphylaxis confirmed by an elevated tryptase serum level.

**Table 2:** Reported cases of RISS with concomitant or subsequent anaphylaxis CRP: C-reactive protein; MALT: Mucosal associated lymphoma tissue; NA: Not available; RA: Rheumatoid arthritis; C: Complement component, RISS: Rituximab-induced serum sickness; RTX: Rituximab; y-o: Years-old

Reference	Background	RTX regimen (dose, course, infusion number in course)	Clinical presentation	Blood Tests	Treatment	Evolution
RISS with concurrent hemodynamic instability						
Rampurwala et al., 2010 [10]	51 y-o woman, Waldenström’s macroglobulinemia	375mg/m^2^ 2, 1	From the 8^th^ day: hypotension (systolic blood pressure: 60mmHg), joint pain with swelling of the wrists and knees, fever	CRP 175mg/L, tryptase NA, complement NA	Norepinephrine, vasopressor therapy, systemic steroids	Significant improvement
Cheong et al., 2018 [11]	50 y-o woman, membranous nephropathy	1000mg 1, 2	15 days after the 1^st^ infusion, i.e., 1 day after the 2^nd^ infusion: hypotension (systolic blood pressure: 70mmHg), tachycardia, maculopapular rash, arthralgias, myalgias, fever	CRP 376mg/L, C3 0.63g/L, C4 0.20g/L, tryptase NA	Inotropes stopped within 24 hours, hydrocortisone initially, followed by a 7-day weaning course of prednisolone	Symptoms resolved in 48 hours, no recurrence over 3 months
Current case	86 y-o woman, autoimmune hemolytic anemia with marginal zone lymphoma	375mg/m^2^ 2, 1	From the 6^th^ day: hypotension, maculopapular rash, arthralgias, fever	CRP 530mg/L, C3 0.97g/L, C4 0.04g/L, CH50 12.5IU/mL, tryptase 26.3µg/L, IgG anti-RTX antibodies >100ng/mL	Adrenaline stopped within 2 days, methylprednisolone 2 days, followed by 7 days of tapered prednisone	Resolution of symptoms in 4 days, without recurrence
RISS with subsequent anaphylaxis						
Kumar et al., 2012 [7]	60 y-o woman, seropositive RA	1000mg 2, 1	RISS at the 7^th^ day: erythematous urticarial rash, pain and swelling of shoulders, fever up to 39°C	NA	Methylprednisolone 80mg 1 day	Resolution of symptoms in 3 days
		1000mg 2, 2 (day 15)	Anaphylaxis within minutes of starting the infusion: swelling of the lips and periorbital areas, choking sensation, tachycardia, no hypotension	NA	Discontinuation of RTX infusion, adrenaline	Resolution of symptoms in 1 hour
Isoda et al., 2016 [8]	65 y-o woman, Sjögren’s syndrome MALT lymphoma	375mg/m^2^ 1, 2	RISS after several days: rash, arthralgias, fever	NA	NA	NA
		375mg/m^2^ 1, 3	Anaphylactic reaction	NA	NA	NA
Bayram et al., 2020 [9]	6 y-o child, membranous nephropathy	375mg/m^2^ 1, 1	RISS after 8 days: arthralgias, myalgias	NA	No intervention	Improvement in 1 day
		375mg/m^2^ 1, 2	RISS after one week: rash, arthralgias	NA	No intervention	Resolution of symptoms in 2 days
		375mg/m^2^ 1, 3	Anaphylaxis during infusion: swelling of the lips and periorbital areas, choking sensation, generalized erythematous rash, hemodynamically stable	CRP 0.7mg/L, C3 1.16g/L, C4 0.14g/L	Discontinuation of RTX infusion, methylprednisolone, adrenaline	Rapid resolution of symptoms
Giraud et al., 2021 [4]	Cryoglobulinemic vasculitis	1000mg 1, 1	RISS	NA	NA	NA
		1000mg 2, 1	Anaphylaxis: Quincke’s edema	IgG anti-RTX antibodies 55ng/mL	NA	NA

The temporal sequence and the absence of other identified triggers of anaphylaxis or shock in these cases raise the hypothesis that the immune response triggered by an RTX infusion leading to SS could also, without further stimulation, lead to anaphylaxis. How biochemical mediators leading to SS may trigger anaphylaxis remains unclear. Two pathways could theoretically link type III and type I HSRs: 1) ICs crosslinking fragment crystallizable gamma receptors (FcγRs) which activate macrophages and neutrophils (type III HSR), as well as mast cells and basophils (IgG-mediated type I HSR); 2) C3a and C5a, formed through complement cascade activation by ICs, attracting macrophages and neutrophils (type III HSR) and triggering mast cells and basophils degranulation (complement-mediated type I HSR) [12,13]. Indeed, IgG-related anaphylaxis is supposed to occur when a large amount of the causative antigen is administered intravenously, such as during an RTX infusion, and a larger amount of antibody is present [12]. Also, delayed IgE-related anaphylaxis could result from an initial under-representation of IgE antibodies blocked by IgG antibodies forming ICs with the antigen which would then exhaust with the SS [1,12]. The first two hypothesis seems the most consistent as anti-RTX IgG antibodies remained high after the sudden hypotensive episode in our case. Reports of RISS subsequently followed by anaphylaxis could as well be the result of either an IgG and complement-mediated anaphylaxis or an IgE-mediated anaphylaxis unmasked after anti-RTX IgG antibodies exhaustion or following the formation of anti-RTX IgE antibodies between courses [4,5,12].

Hypersensitivity reactions raise safety issues regarding continued RTX therapy. On the one hand, in most of the reported cases, patients who underwent RTX rechallenge after RISS presented no symptoms [3]. On the other hand, some authors recommend that RTX should not be rechallenged after SS [5]. The few reports of RISS followed by anaphylaxis on subsequent infusion support the latter statement [4,7-9]. Finally, there are several ways to overcome type I HSR, namely dose and infusion rate reduction, administration of prophylactic fluid (normal saline up to 500mL/h) to dilute the causative antigen, and desensitization [4,5]. Regardless of the choice made (rechallenging RTX or switching to another drug when available [4]), treatment must be individualized and discussed in a multidisciplinary meeting to ensure the best benefit/risk ratio.

## Conclusions

Anaphylaxis can be a rare and severe but reversible complication of RISS. This condition could result from a combination of complement consumption, elevated serum tryptase, and the presence of anti-RTX antibodies. Hemodynamic instability occurring with RISS should prompt the administration of epinephrine with a dosage of complement, tryptase, and anti-RTX antibodies. Modalities of rechallenge should be discussed on a case-by-case basis.
